# Using precision tools to manage and evaluate the effects of mineral and protein/energy supplements fed to grazing beef heifers

**DOI:** 10.1093/tas/txad013

**Published:** 2023-01-28

**Authors:** Kacie L McCarthy, Sarah R Underdahl, Michael Undi, Carl R Dahlen

**Affiliations:** Center for Nutrition and Pregnancy and Department of Animal Sciences, North Dakota State University, Fargo, ND, USA; Center for Nutrition and Pregnancy and Department of Animal Sciences, North Dakota State University, Fargo, ND, USA; Central Grasslands Research Extension Center, North Dakota State University, Streeter, ND, USA; Center for Nutrition and Pregnancy and Department of Animal Sciences, North Dakota State University, Fargo, ND, USA

**Keywords:** activity monitoring, beef cattle, electronic feeder, mineral, supplement, grazing

## Abstract

Our objectives were to develop a Mobile Cow Command Center (MCCC) capable of precision monitoring of grazing heifers to 1) examine the relationship between supplement intake on concentrations of liver mineral and blood metabolites and 2) examine activity, reproductive, and health behavior. Yearling crossbred Angus heifers (*N* = 60; initial BW = 400.4 ± 6.2 kg) were fitted with radio frequency identification ear tags that allowed access to electronic feeders (SmartFeed system; C-Lock Inc., Rapid City, SD), and with activity monitoring tags (CowManager B.V., the Netherlands) that monitored reproductive, feeding, and health-associated behaviors. Heifers were assigned randomly to one of three treatments for a 57-day monitoring period: 1) no supplement (**CON**; *N* = 20), 2) free choice mineral (**MIN**; Purina Wind and Rain Storm [Land O’Lakes, Inc.], *N* = 20), or 3) free choice energy and mineral supplement (**NRG**; Purina Accuration Range Supplement 33 with added MIN [Land O’Lakes, Inc.], *N* = 20). Consecutive day body weights, blood, and liver biopsies were collected at pasture turnout and final day of monitoring. By design, mineral intake was greatest in MIN heifers (49 ± 37 g/d) and energy supplement intake was greatest in NRG heifers (1,257 ± 37 g/d). Final BW and ADG were similar among treatments (*P* > 0.42). Concentrations of glucose on day 57 were greater (*P* = 0.01) in NRG compared with CON and MIN heifers. Liver concentrations of Se and Fe on day 57 were greater (*P* < 0.05) in NRG heifers than CON, with MIN being intermediate. Activity tags reported NRG heifers spent less time eating (*P* < 0.0001) and more time (*P* < 0.0001) being “highly active” than MIN with CON heifers being intermediate. Data retrieved from activity tags identified 16 of 28 pregnant heifers exhibiting some type of estrus-associated behavior even after confirmation of established pregnancy. The activity monitoring system triggered a total of 146 health alerts from 34 of the 60 heifers monitored, but only 3 heifers of the heifers initiating an electronic health alert needed clinical treatment. However, animal care staff identified nine additional heifers that required treatment for which no electronic health alert was generated. The electronic feeders successfully controlled intake of individual heifers managed in groups pastures; however, the activity monitoring system misrepresented estrus and health events.

## INTRODUCTION

Technology continues to improve and some sectors of agriculture are rapidly implementing new innovations into diverse applications. The beef industry, however, is slower than other agricultural industries in rate of adoption (Dahlen et al., 2014; Lamb et al., 2016). Several reasons likely exist for this adoption lag, foremost of which are the lack of comprehensive technological solutions that can be implemented in expansive pasture settings, and the lack of solutions from which management decisions can be made over the life of an animal. Individual animals within a herd of cattle are unique, are in varying stages of production, have specific nutritional needs, and present differing health statuses. Within the herd, individual animal variation exists and changes throughout the production year, presenting real and relevant management issues for progressive producers.

Producers often provide mineral and/or protein and energy supplements to grazing cattle to maintain targeted production goals for growth and reproductive performance (Schillo et al., 1992; Ciccioli et al., 2005; Cappellozza et al., 2014) and to offset forage nutritive decline throughout the grazing season (Schauer et al., 2004; Cline et al., 2009; McCarthy et al., 2021). An issue observed with providing supplements on pasture is the large variability in consumption by individuals within a group (Tait and Fisher, 1996; Bowman and Sowell, 1997; Cockwill et al., 2000; Patterson et al., 2013), which is largely unseen and unknown by cattle management personnel. In addition, frequent observation of activity and reproductive behavior of grazing cattle is often difficult due to the expansive nature of pastures and being labor intensive (Elischer et al., 2013). Electronic systems that can monitor feeding, physical activity, and reproductive-related behavior are now available.

Activities reported in this study are aimed at developing a system (the Mobile Cow Command Center) that pairs multiple technologies into a single portable unit that would allow for precision management of individuals within a herd on expansive pastures to optimize production efficiency, improve animal health, and enhance profitability. Our objectives were to develop a Mobile Cow Command Center (MCCC) for monitoring heifers on native range specifically to 1) examine the relationship between mineral and energy supplementation on intake, liver mineral concentrations, and metabolites and 2) examine activity, reproductive, and health behavior.

## MATERIALS AND METHODS

All animal procedures were approved by the Institutional Animal Care and Use Committee at North Dakota State University (A18069).

### Study Area

Research was conducted at the Central Grasslands Research Extension Center (CGREC), located near Streeter, ND from July 25 to September 19. This area is characterized by a continental climate with warm summers and cold winters with a majority (72%) of precipitation occurring between May and September (Limb et al., 2018). August is the warmest month with a mean temperature of 18.6°C (NDAWN, 2017).

The pasture was 70 ha with a stocking rate of 1.99 Animal Unit Months (AUMs)/ha. The vegetation is classified as mixed-grass prairie dominated by western wheatgrass (*Pascopyrum smithii* [Rydb.] À. Löve), green needlegrass (*Nassella viridula* [Trin.] Barkworth) and blue grama (*Bouteloua graciles* [Willd. ex Kunth] Lag. ex Griffiths). Other important species include sedges (*Carex* spp.), prairie junegrass (*Koeleria macrantha* [Ledeb.] Schult.), sages (*Artemisia* spp.), goldenrods (*Solidago* spp.), kentucky bluegrass (*Poa pratensis* L.) a nonnative grass and western snowberry (*Symphoricarpos occidentalis* Hook.) a native shrub (Limb et al., 2018).

### Mobile Cow Command Center Units

Each of two Mobile Cow Command Center (MCCC) units were developed by pairing two commercially available technologies into single trailer units that can be transported and function anywhere cattle are managed. The first technology is the SmartFeed device (C-lock Inc., Rapid City, SD), which is a self-contained system designed to measure supplement intake and feeding behavior from individual cattle in group settings. The system is solar powered and includes a radio-frequency identification (RFID) reader, weigh scales, access control gate, a feed bin, and a cloud-based interface which continuously logs feed intake and feeding behavior data. The programming of the SmartFeed units is flexible, with the ability to assign specific animals to specific feeders and to prohibit entry of individual animals once a daily target intake is achieved. The second technology included in the MCCC was the CowManager system (CowManager B.V., the Netherlands), which fits over an RFID ear tag and uses additional sensors to monitor cow reproductive (estrus alerts), feeding-related (eating, rumination, and activity level), and health-associated data. The CowManager ear tag continuously registers movements from the cow’s ear and classifies the data through proprietary algorithms (Pereira et al., 2018). Data are sent through a wireless connection, via a router placed on the top of the MCCC unit. Data are then received through a coordinator unit that is attached to a computer in a lab (approximately 200 m line of site from the MCCC units) that automatically uploaded the data for viewing on any device with an internet connection. Each MCCC contained 2 SmartFeed units, controlling hardware and the CowManager router in an enclosed trailer with open feed access areas and retractable wheels for transport.

### Training Period

Two heifer development pens (*N* = 63 per pen) were utilized at the CGREC for a 2-week training period where one MCCC unit was placed in each dry lot pen. A portion of the heifer development ration (corn silage) was placed into the feed bins and heifer intake was monitored. Only heifers with a history of feed consumption from the feeders were selected as experimental units for this experiment.

### Heifer Selection

All heifers were estrus synchronized using a controlled internal drug release (CIDR; Zoetis, Parsippany, NJ) protocol (7 d CO-Synch plus CIDR), with heifers receiving 2 mL intramuscularly GnRH (Factrel; Zoetis, Parsippany, NJ) and CIDR insert on day 0. Seven days later, the CIDR insert was removed and a single injection of PGF_2α_ (5 mL intramuscularly; Lutalyse; Zoetis, Parsippany, NJ) were administered followed by GnRH and artificial insemination (AI), approximately 60 h later. All heifers received an estrus detection patch (Estrotect; Rockway Inc., Spring Valley, WI) to monitor estrus (Hill et al., 2014). On the day of AI, final heifer selection for the experiment was made based on 1) history of consuming feed from SmartFeed feeders and 2) activated estrus detection patches. A total of 60 of the 126 heifers met the criteria for inclusion in the experiment and were bred using sexed semen (Tehama Tahoe B767 14AN502) for female offspring.

### Grazing Period

**Figure 1.**
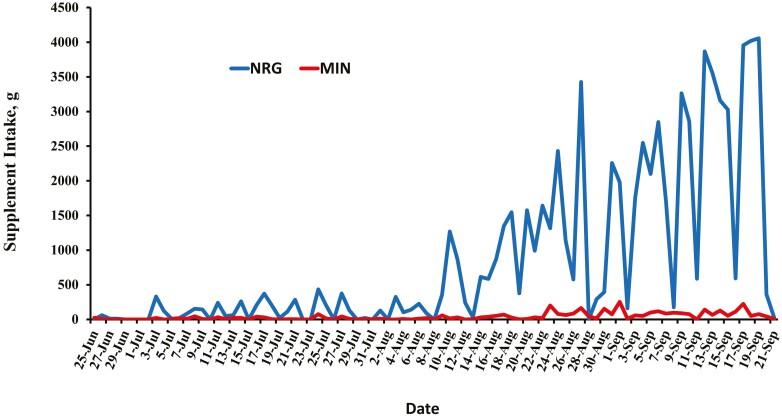
Daily intake of mineral (MIN) or energy with mineral (NRG) supplements of heifers grazing native range over the duration of the grazing season. The 57-d monitoring period was initiated from the time of pregnancy diagnosis (July 25th) until removal from pasture (September 19th). Treatments include: MIN (*N* = 18), free choice access to mineral supplement [Purina Wind & Rain Storm All-Season 7.5 Complete (Land O’Lakes, Inc., Arden Hills, MN)]; NRG (*N* = 13), free choice access to energy supplement [Purina Accuration Range Supplement 33 with added MIN (Land O’Lakes, Inc., Arden Hills, MN)].

Sixty crossbred yearling Angus heifers (initial BW = 400 ± 6 kg) were managed as a single pasture group with free access to graze native range and were randomly assigned to 1 of 3 dietary treatments 1) no access to feed supplements (CON; *N* = 20), 2) free choice access to mineral supplement (MIN; Purina Wind & Rain Storm All-Season 7.5 Complete, Land O’Lakes, Inc., Arden Hills, MN, *N* = 20), or 3) free choice access to energy supplement (NRG; Purina Accuration Range Supplement 33, Land O’Lakes, Inc., Arden Hills, MN, *N* = 20). The manufacturer recommendation for daily intake of the mineral supplement was 113 g. The NRG supplement was formulated by adding 68.1 kg MIN to a 907.4 kg mixture of 60% ground corn and 40% Accuration (25.5 % CP; Table 1) with an anticipated daily intake of 1.63 kg. Thus, if heifers in the NRG treatment consumed 1.63 kg of supplement, and heifers in the MIN treatment consumed 113 g, then both the MIN and NRG heifers would be consuming the same amount of the mineral product used. The MIN and NRG supplements were delivered via the MCCC units which were located within 50 m of the waterer in the pasture. Feeders were set to restrict access of CON heifers from either trailer unit, with MIN and NRG heifers having ad libitum access to the trailer containing their respective feed assignment. Because few heifers consumed either supplement early in the grazing season (Figure 1), feed intake data were summarized over a 57-d period; from the time of pregnancy diagnosis (July 25) until removal from pasture (September 19).

**Table 1.** Dietary ingredient and nutrient composition of mineral (MIN) and energy with mineral (NRG) supplement fed to grazing beef heifers

	% DM basis	
Nutrient analysis	NRG^1^	MIN^2^
DM	94.95	—
Ash	12.69	—
CP	25.49	—
N	4.08	—
NDF	15.77	—
ADF	5.78	—
Ether extract	6.17	—
Mineral analysis, mg/kg		
Ca	18,499	176,939
P	10,047	76,274
S	7,150	8,165
Se^3^	<100.0	<100.0
Fe	462	6,628
Cu	1,079	796.3
Zn	429.9	2,590.5
Mo	8.6	15.7
Mn	202.6	2,860.4
Co	67.14	10.35

^1^NRG = Purina Accuration Range Supplement 33 with added MIN (Purina Wind & Rain Storm All-Season 7.5 Complete; Land O’Lakes, Inc., Arden Hills, MN). Formulated by adding 68.1 kg MIN to a 907.4 kg mixture of 60% ground corn and 40% Accuration.

^2^MIN = Purina Wind & Rain Storm All-Season 7.5 Complete (Land O’Lakes, Inc., Arden Hills, MN). Ingredients: Dicalcium Phosphate, Monocalcium Phosphate, Calcium Carbonate, Salt, Processed Grain ByProducts, Vegetable Fat, Plant Protein Products, Potassium Chloride, Magnesium Oxide, Vitamin E Supplement, Vitamin A Supplement, Natural and Artificial Flavors, Calcium Lignin Sulfonate, Ethoxyquin (a Preservative), Manganese Sulfate, Vitamin D3 Supplement, Zinc Sulfate, Basic Copper Chloride, Ethylenediamine Dihydroiodide, Cobalt Carbonate. The vitamin was labeled to contain 136,054, 13,605, and 136 IU/kg of Vitamins A, D, and E, respectively.

^3^Analysis for concentrations of trace minerals was done using an ICP-OES panel for premix evaluation with the lowest detection limit for Se of 100 mg/kg. Company guaranteed analysis for concentrations of Se in MIN supplement was 27 mg/kg.

The CowManager system reported the minutes spent during each hour of every day in activity categories including “eating”, “ruminating”, “not active”, “active”, and “highly active”, with a proprietary model and available through the web-based application. Estrus-related alerts were continuously generated via the CowManager system, including classifications of “in heat”, “potential”, or “suspicious”. Pregnancy detection was performed 34 d after AI via transrectal ultrasonography (7.0-MHz transducer, 500 V Aloka, Wallingford, CT). Continuous monitoring with the CowManager tag provided data related to heifer estrus activity. A retrospective analysis was conducted to determine the accuracy of estrus-related alerts generated via the CowManager system versus a known pregnancy status determined via ultrasound. Similarly, a retrospective analysis was conducted to evaluate the accuracy of health events that were flagged via the CowManager system (reported as “sick”, “very sick”, or “no movement”) by comparing electronic alerts with treatment logs generated by the animal care staff. It is important to note that the CowManager system has been validated using the proprietary algorithm in populations of dairy cows housed indoors (Bikker et al., 2014) and grazing (Pereira et al., 2018).

### Forage Collection and Analysis

Forage samples were obtained every 2 weeks from 20 different locations in the pasture in a diagonal line across the pasture. The forage samples were hand clipped to a height of 3.75 cm above ground (Undi et al., 2008). Forage samples were dried in a forced-air oven at 60°C for at least 48 h and then ground to pass through a 2-mm screen using a Wiley mill (Arthur H. Thomas, Philadelphia, PA). Clipped forage samples for each location reported herein were composite over all locations within the representative sampling date and reported as averages within month. Forage samples were analyzed at the North Dakota State University Nutrition Laboratory for dry matter (DM), ash, N (Kjehldahl method), Ca, P, and ether extract (EE) by standard procedures (AOAC, 1990). Crude protein (CP) was calculated by multiplying N by 6.25. Neutral detergent fiber (NDF) and acid detergent fiber (ADF) concentrations were determined by the modified method of Van Soest et al. (1991) using a fiber analyzer (Ankom Technology Corp., Fairport, NY). Samples were also analyzed for Cu, Zn, Co, Mo, Fe, S, and Se using inductively coupled plasma optical emission spectroscopy (ICP-OES) by the Veterinary Diagnostic Laboratory at Michigan State University.

### Liver Sample Collection and Analysis

Because the liver is a major organ of mineral storage and concentrations of minerals in the liver are indicative of mineral status in ruminants (Spears et al., 2022), liver samples were collected at pasture turnout and at the final day of monitoring via biopsy from a subset of heifers from each respective treatment (*N* = 24, 8 per treatment). Liver biopsy samples (approximately 20 mg) were collected as previously described by McCarthy et al. (2020). Liver samples frozen at −20°C, then sent on ice to the Veterinary Diagnostic Laboratory at Michigan State University and were evaluated for concentrations of minerals using inductively coupled plasma mass spectrometry (ICP-MS).

### Blood Collection and Serum Analysis

Blood metabolites were analyzed from a subset of heifers from each respective treatment (*N* = 30). Blood samples were collected at pasture turnout and at the final day of monitoring via jugular venipuncture into serum tubes (10 mL; Becton Dickinson Co., Franklin Lakes, NJ), allowed to clot for 30 min and centrifuged at 1,500 × *g* at 4°C for 20 min. Serum was separated and stored in plastic vials at −20°C until further analysis. Serum samples were analyzed for glucose and NEFA. Samples were analyzed using the Synergy H1 Microplate Reader (Biotek, Winooski, VT) with the Infinity Glucose Hexokinase Kit (Thermo Scientific, Waltham, MA) and NEFA-C Kit (WAKO Chemicals, Inc., Richmond, VA). The intra- and interassay CV was 2.62% and 3.41%, for serum glucose, respectively and 7.75% and 8.29%, for serum NEFA, respectively.

### Statistical Analysis

Heifers assigned to MIN and NRG treatments that did not voluntarily consume their assigned supplements from the electronic feeders were retrospectively added to CON treatment for analysis, resulting in a final *N* of 29 CON, 18 MIN ,and 13 NRG heifers, respectively. Data were analyzed as a completely randomized design with heifer used as the experimental unit for all analysis. Performance and intake data were analyzed using the GLM procedure of SAS (9.4, SAS Inst. Inc., Cary, NC) with treatment as the fixed effect. Blood metabolites were also analyzed using the GLM procedure and the model statement used contained the effects of treatment and baseline serum metabolite concentrations at pasture turnout. Concentrations of mineral in liver samples were analyzed using the GLM procedure and the model statement used contained the effect of treatment, with values from baseline pasture turnout liver samples used as a covariate. Total mineral intake from supplemental sourced was calculated by multiplying total supplement intake over the 57-day monitoring period by the analyzed concentration of the respective supplement (MIN or NRG) consumed by individual heifers. Total mineral intake data were analyzed using the GLM procedure of SAS with a model including treatment. Data for activities including daily time spent eating, ruminating, not active, active, and highly active were analyzed using the MIXED procedure of SAS for repeated measures in time with treatment, day, and their interaction in the model. Results are reported as least square means using the LSMEANS statement for liver and plasma. For all analysis, significance was set at *P* ≤ 0.05.

## RESULTS AND DISCUSSION

### Heifer Intake, Feeding Behavior, and Performance

Intake of energy and mineral supplements were minimal during the early portion of the grazing season but began to increase in midAugust as the quality of native range declined (Figure 1). Proportion of days attending feeders was greater (*P* < 0.001) for NRG heifers (68 ± 2.04%) compared with MIN heifers (41 ± 2.04%). Overall number of days heifers were present at the NRG and MIN feeders was 38.7 and 23.1 ± 1.2 d of the 57 d, respectively. More supplement (*P* < 0.001) was consumed on days that NRG heifers attended feeders (1,877 ± 76 g/d of energy supplement) compared with days when MIN heifers attended feeders (122 ± 76 g/d of mineral supplement). Energy supplement heifers spent more (*P* = 0.01) time at the feeders (4.1 ± 0.6 min/d) on days they attended feeders compared with MIN (2.1 ± 0.6 min/d) and CON heifers (1.3 ± 0.6 min/d). Additionally, NRG heifers visited the feeders more (*P* < 0.001) times (9.20 ± 0.34 times/d) on days they attended feeders compared with MIN (2.71 ± 0.34 times/d) and CON heifers (1.94 ± 0.34 times/d).

Over the 57-day monitoring period, heifers in the MIN treatment consumed 49.3 ± 37 g/d of mineral supplement. Heifers in the NRG treatment consumed 1,257.1 ± 37 g/d of energy supplement. Mean values for NRG supplement intake by heifers in the CON treatment were driven by 3 heifers that consumed a total of 63.3 g/d over the monitoring period at the NRG supplement feeder. Mean values for MIN supplement intake by heifers in the CON treatment were driven by 26 feeding attempts where 2.8 g/d of MIN supplement was consumed during 7.7 of the 57 d (13% attendance) during the monitoring period. Over the monitoring period, NRG heifers spent more (*P* < 0.001) time (2.9 ± 0.34 min/d) at the feeder compared with MIN (0.72 ± 0.34 min/d) and CON heifers (0.18 ± 0.34 min/d). Additionally, during the monitoring period, NRG heifers visited the feeders more (*P* < 0.001) times daily (6.18 ± 0.15 times/d) than MIN (2.71 ± 0.15 times/d) and CON heifers (0.25 ± 0.15 times/d).

Certainly, heifers not assigned to the respective treatments did attempt to consume supplement. However, the SmartFeed system was able to limit the frequency of feeder attendance and supplement consumption by heifers that were not designated to consume the respective supplements via that online control system. Cows that were being fed in SmartFeed units (McCarthy et al., 2021) consumed more mineral supplement on average (125.4 g/d) than heifers reported herein. In comparison, Smith et al. (2016) built a custom mineral feeder with an RFID reader and reported that steers that had access to a commercially available free-choice mineral consumed 72 g/d per head over a 90-d grazing period. The mineral disappearance in Smith et al. (2016) was within a range of manufacturer recommended intakes of 40 to 125 g/head per day. Moreover, researchers in Oklahoma (Reuter et al., 2017) conducted a pilot study using the SmartFeed system to characterize the daily variation in soybean meal supplement with the inclusion of salt on intake by group-housed, self-fed grazing steers. Fifteen steers from Reuter et al. (2017) consumed 1,210 g/d of supplement with a 45% salt inclusion for a 14-d period. Although steers consumed a similar amount of supplement compared to heifers reported herein, variation among animals was also reported with animals visiting 5.1 ± 1.3 times/d over the 14-d period (Reuter et al., 2017). Over the duration of this study, heifers visited the feeders a similar number of times compared with those reported by Reuter et al. (2017) utilizing the same feeder technologies. The variation among animals from Reuter et al. (2017) suggests that competition for use of one SmartFeed unit may have been a challenge because intervals between different RFID readings (animals exchanging places at the feeder) was less than 1 s per animal. Heifers in the current study may have been experiencing similar challenges with competition at the feeder even though they had an additional SmartFeed unit to visit. This postulate was corroborated with visual observations of heifers vigorously exchanging places at the feeder when the herd did visit the proximity of the feeders.

The manufacturer label for the mineral supplement provided recommended optimum intakes of 113 g/head daily. Over the 57-day monitoring period, 1 in 18 (0.06%) MIN heifers consumed recommended MIN intake, but heifers did not attend feeders daily. On days they did attend the feeder 10 of the 18 (56%) heifers that attended the feeders consumed recommended feeding rates of MIN supplement. Variation in individual consumption has been related to number and placement of feeders, individual animal preference, weather, individual and herd behavior, characteristics of the feedstuff, and feed additives that may be included (Tait and Fisher, 1996; Bowman and Sowell, 1997; Smith et al., 2016).

Overall, heifer final BW was similar among treatments (433 ± 6 kg; *P* = 0.42). Interestingly, treatment did not influence body weight gain (*P* = 0.76) during the monitoring period, with heifer ADG equal to 0.46 kg/d. Many studies highlight enhanced gain in heifers consuming supplemental feeds (Delcurto et al., 2000; Engel et al., 2008; Summers et al., 2015). However, previous studies have reported that neither trace mineral supplementation nor source (organic and inorganic) affected cow BW or BCS (Olson et al., 1999; Muehlenbein et al., 2001; Ahola et al., 2004). Similarly, pregnant heifers provided an energy supplement as a mixture of cracked corn, soybean meal, and urea while consuming low-quality cool-season forages in feedlot pens reported similar ADG (0.75 kg/d) over a 19-day feeding period (Cappellozza et al., 2014a). Heifers on the NRG treatment in the current experiment were likely substituting a portion of forage intake with the supplement consumed (Summers et al., 2015). Collectively, heifers assigned to the NRG treatment may have simply not consumed enough supplement over the course of the experiment to compensate for reduced forage intake and elicit a subsequent gain response.

### Blood Metabolites

Though no gain response was observed, concentrations of glucose in serum were 14% greater (*P* = 0.01) in NRG heifers compared with CON and MIN heifers at the end of the monitoring period (Table 2). Similar concentrations of glucose have been reported in beef heifers offered low-starch energy supplements daily or three times weekly (76.3 and 70.5 mg/dL, respectively; Moriel et al., 2012) or where heifers received either energy (provided as cracked corn, soybean meal, and urea) or protein (provided as soybean meal) supplements while consuming cool-season forages (65.0 and 65.1 mg/dL, respectively; Cappellozza et al., 2014b). Since starch is a major dietary precursor for glucose in ruminants (Huntington, 1997), the observation of elevated concentrations of glucose in heifers receiving the NRG (i.e. starch-based) supplement was expected. Starch fermentation in the rumen results in greater propionate and less acetate production, and therefore a greater supply of glucose to the animal (Huntington, 1997). Furthermore, other studies (Cooke et al., 2008) have reported increases in plasma glucose concentration in heifers supplemented infrequently and attributed those increases to the time required for synthesis and activation of gluconeogentic enzymes to change glucose synthesis and released by the liver.

**Table 2.** Effects of mineral (MIN) or energy with mineral (NRG) supplements on concentrations of serum metabolites in heifers grazing native range

	Treatment^1^				*P*-value
Item^2^	CON	MIN	NRG	SEM	TRT
NEFA, µmol/L	327.1	326.2	291.7	47.04	0.85
Glucose, mg/dL	66.7^b^	66.5^b^	75.9^a^	2.12	0.01

^a,b^Means within a row with a different superscript differ (*P* < 0.05).

^1^Treatments include: CON (*N* = 12), no access to feed supplements; MIN (*N* = 10), free choice access to mineral supplement [Purina Wind & Rain Storm All-Season 7.5 Complete (Land O’Lakes, Inc., Arden Hills, MN)]; NRG (*N* = 8), free choice access to energy supplement [Purina Accuration Range Supplement 33 with added MIN (Land O’Lakes, Inc., Arden Hills, MN)].

^2^Results covariately adjusted to baseline serum sample taken at pasture turnout.

There were no differences among treatments in concentrations of NEFA in serum at the conclusion of the experiment (*P* = 0.85; Table 2). Circulating NEFA concentrations reflect fat mobilized from body reserves, with elevated concentrations often associated with negative energy balance. Nevertheless, it is important to note that heifers from all treatments were in a positive nutritional status based on similar ADG and, therefore, no mobilization of body reserves was likely necessary in any treatments. As animals experience compensatory gain, concentrations of NEFA have been reported to rapidly decline (Ellenberger et al., 1989). McFarlane et al. (2017) provided protein supplement to growing heifers grazing winter forage and reported no differences in concentrations of NEFA in serum, which was not expected due to the fact that the authors observed BW changes in heifers. In contrast, Cappellozza et al. (2014b) reported that control heifers had greater concentrations of NEFA compared with heifers receiving either energy (provided as cracked corn, soybean meal, and urea) or protein (provided as soybean meal) supplements while consuming cool-season forages. Although it is important to note that Cappellozza et al. (2014b) reported unexpected differences with control heifers, all heifers were in a positive nutritional status. Therefore, lack of differences among treatments reported herein was likely due to heifers not being in negative energy balance through the grazing period.

It is important to note that during the time of supplementation in the current study, heifers were in early stages of gestation. The maternal gastrointestinal tract is critical for nutrient acquisition and is a major nutrient sink during pregnancy (Vonnahme et al., 2015) and the relationship between maternal nutrient intake during pregnancy and fetal growth are extremely important (Redmer et al., 2004; Dahlen et al., 2021; Reynolds et al., 2022). Research from Perry et al. (1999) determined that low dietary protein (provided as cottonseed meal) in the first trimester of pregnancy followed by increased protein in the second trimester may have an effect on placental development and thus subsequent impacts on calf body weight in primiparous heifers. In addition, a similar mineral and protein/energy supplements fed to beef heifers during early gestation resulted in altered fetal liver and femur weights, concentrations of mineral in fetal liver and muscle, concentrations of amino acids in the amniotic fluid, and altered placental gene expression by day 83 of gestation (Diniz et al., 2021; Menezes et al., 2021; McCarthy et al., 2022; Menezes et al., 2022). Therefore, observations of altered metabolite and mineral profiles as observed in the current experiment may be impacting the developing fetus, and further investigation is warranted to determine the potential of using electronic feeding equipment to impose developmental programming effects on offspring conceived and gestated in extensive pasture conditions.

### Forage Analysis

Forage nutrient content appeared to decrease over the course of the grazing period (Table 3) observed by percentage of CP decreasing and greater NDF values over the season. As forage nutritive value decreased, we observed increases in supplement intakes (Figure 1). A decrease in the forage nutritive value is typical in diets of grazing cattle during the advancing season (Bedell, 1971; Johnson et al., 1998; Cline et al., 2009; McCarthy et al., 2021). Typically, the nutrient availability of grazed forages fluctuates by environmental conditions, forage species, soil type and stage of maturity (NASEM, 2016).

**Table 3.** Forage analysis of representative sample composites of pasture grazed by beef heifers provided either mineral (MIN) or energy with mineral (NRG) supplement from June to September^1^

	Grazing period^2^			
Item	June	July	August	September
TDN^3^	60.5	62.0	60.6	58.6
CP, %	9.02	7.1	6.8	5.9
Ash	10.04	9.4	10.3	10.5
NDF, %	62.87	59.1	61.1	64.5
ADF, %	35.9	34.1	35.9	38.4
Ca, %	0.21	0.33	0.41	0.42
P, %	0.40	0.14	0.12	0.10
S, %	0.1416	0.1498	0.1616	0.1503
Se, mg/kg	<10.0	<10.0	<10.0	<10.0
Fe, mg/kg	<50	101	130	166
Cu, mg/kg	4.6	4.1	4.5	3.8
Zn, mg/kg	14.8	17.7	20.0	23.7
Mo, mg/kg	1.4	1.4	1.7	1.3
Mn, mg/kg	59	60.7	84.0	100.4
Co, mg/kg	<1.00	<1.00	<1.00	<1.00

^1^Clipped forage samples from 20 different locations reported herein are composite over all locations within the representative sampling dates.

^2^Values presented are mean values of the representative sampling dates within the given month: June (*N*= 1), July (*N* = 3), August (*N* = 2), and September (*N* = 3).

^3^TDN = 88.9 – (0.79 × ADF%); Holland and Kezar, 1995

For beef breeding cattle, recommended allowances for Se, Fe, Cu, Zn and Mn are 0.10, 50, 10, 30 and 40 mg/kg of diet, respectively (NASEM, 2016). Iron in pastures has been shown to have seasonal fluctuations with peaks in spring and autumn (Suttle, 2010), where our current forage Fe concentrations are greater over the course of the grazing season. According to Corah and Dargatz (1996), forage Fe is within adequate levels at 50 to 200 mg/kg. Most forage contains 70 to 500 mg Fe/kg (NASEM, 2016), which the current pasture falls within the range. Concentrations of Cu in forage were marginal to deficient (4 to 7 vs. < 4 mg/kg, respectively; Corah and Dargatz, 1996). Forages vary in Cu content, with legumes usually having higher content than grasses (NASEM, 2016). Moreover, concentrations of Zn were deficient (<20 mg/kg) until midAugust to early September. According to Corah and Dargatz (1996), Mo, Co, and Mn were adequate (<1, 0.1 to 0.25, and >40 mg/kg, respectively). As stated by Suttle (2010), Mn values for pastures vary, with a mean value of 86 mg/kg. In addition, Mn requirements for breeding cattle are higher than growing and finishing cattle due to reproduction demands (NASEM, 2016).

Similar forage responses in the Northern Great Plains have been reported by investigators (Johnson et al., 1998; Cline et al., 2009) who have observed increases in forage fiber content with the advancing season. In addition, available forage protein may decrease enough over the grazing season that optimal livestock performance may require supplementation (Bodine et al., 2001). As noted, intakes of NRG supplement by heifers increased with advancing season. Furthermore, mineral intake is often affected by season of the year, with the greatest intakes often during the winter or dry season when forages stop growing, become high in fiber and lignin and low in digestibility (McDowell, 1996). This was also corroborated with MIN supplemented heifers that started consuming more supplement later in the season. Moreover, native forages typically grazed by beef cattle are generally deficient to marginal in Cu, Mn, Se, and Zn concentrations (Umoh et al., 1982); therefore, supplying supplemental minerals under these grazing conditions are typically performed. Over the course of the grazing season, the most notable change in forage mineral concentration were noted in decreasing concentrations of Cu, and increasing concentrations of Fe, Zn, and Mn.

### Liver Mineral Concentrations and Supplemental Mineral Intake

At the end of the monitoring period, concentrations of Se and Fe in liver of NRG heifers were greater (*P* = 0.01; Table 4) than CON, whereas MIN were intermediate. Concentrations of liver Co at the end of the monitoring period were greater in NRG heifers (*P* < 0.001) compared with MIN, which were greater (*P* < 0.001) than CON. Furthermore, no differences (*P* ≥ 0.12) were observed in concentrations of liver Cu, Zn, Mo, and Mn among treatments. According to guidelines published by Kincaid (2000), liver concentrations of Fe, Zn, Se, Mo, and Mn in all treatment groups were considered adequate at the end of the grazing period. Additionally, concentrations of liver Co in all treatment groups were above levels considered to be satisfactory (0.08 to 0.12 μg/g DM; McNaught, 1948). In contrast, Cu values would be considered marginal (33 to 125 μg/g DM; Kincaid, 2000). The lower concentrations of Cu noted in CON heifers can be supported by the low forage value for Cu concentrations reported herein and therefore mineral supplementation may result in increased concentrations of Cu in heifers grazing native range.

**Table 4.** Effects of mineral (MIN) or energy with mineral (NRG) supplements on liver mineral concentrations at pasture removal in heifers grazing native range

	Treatment^1^				*P*-value
Item^2^, µg/g	CON	MIN	NRG	SEM	TRT
Se	1.40^b^	1.61^a,b^	1.85^a^	0.118	0.01
Fe	197.7^b^	213.0^a,b^	286.0^a^	28.57	0.05
Cu	75.3	106.0	110.2	16.39	0.12
Zn	99.8	103.3	112.6	8.60	0.47
Mo	3.65	3.93	3.70	0.269	0.58
Mn	9.25	8.99	10.66	0.810	0.27
Co	0.13^c^	0.32^b^	0.41^a^	0.02	<0.001

^a,b^Means within a row with a different superscript differ (*P* < 0.05).

^1^Treatments include: CON (*N* = 12), no access to feed supplements; MIN (*N* = 7), free choice access to mineral supplement [Purina Wind & Rain Storm All-Season 7.5 Complete (Land O’Lakes, Inc., Arden Hills, MN)]; NRG (*N* = 5), free choice access to energy and mineral supplement [Purina Accuration Range Supplement 33 with added MIN (Land O’Lakes, Inc., Arden Hills, MN)]

^2^Results covariately adjusted to baseline liver biopsy taken at pasture turnout.

Data in the current report align with McDowell (1996) who suggested that the most efficient method for providing supplemental minerals may be through a combination with concentrates to ensure that adequate intake of minerals along with other nutrients are consumed. This strategy can be effective in decreasing the variability in free-choice trace mineral intake and bolstering tissue stores during winter or when energy/protein supplements are provided. The NRG treatment in the current experiment was formulated so that at a projected intake level, heifers would consume the same 113 g/head daily of the same mineral source the MIN heifers were receiving. Though NRG heifers did not achieve the anticipated intake, they ultimately consumed greater (*P* < 0.0001; Table 5) amounts of supplemental Fe, Cu, Zn, Mo, MN, and Co compared with MIN heifers that just had free choice access to the mineral supplement. Therefore, increased liver concentrations of Co for NRG heifers compared with MIN heifers were not unexpected. Also, NRG heifers consumed 77.96% more Fe compared to total supplemental mineral intake in MIN heifers, therefore corroborating the increased concentrations of Fe in the liver of NRG heifers. In addition, because a majority of NRG consumption was late in the monitoring period, we anticipate that if heifers were allowed to continue their respective treatments for additional time that concentrations of other liver minerals would subsequently diverge between the MIN and NRG heifers.

**Table 5.** Total supplemental mineral intake in heifers consuming mineral (MIN) or energy with mineral (NRG) supplements over a 57-d period

	Treatment^1^				*P*-value
Item, g	CON	MIN	NRG	SEM	TRT
Fe	1.1^c^	18.6^b^	33.1^a^	1.96	<0.0001
Cu	0.5^b^	2.2^b^	77.3^a^	2.69	<0.0001
Zn	0.5^c^	7.3^b^	30.8^a^	1.23	<0.0001
Mo	0.005^b^	0.044^b^	0.616^a^	0.022	<0.0001
Mn	0.48^c^	8.04^b^	14.52^a^	0.852	<0.0001
Co	0.026^b^	0.029^b^	4.81^a^	0.167	<0.0001

^a,b^Means within a row with a different superscript differ (*P* < 0.05).

^1^Treatments include: CON (*N* = 12), no access to feed supplements; MIN (*N* = 7), free choice access to mineral supplement [Purina Wind & Rain Storm All-Season 7.5 Complete (Land O’Lakes, Inc., Arden Hills, MN)]; NRG (*N* = 5), free choice access to energy and mineral supplement [Purina Accuration Range Supplement 33 with added MIN (Land O’Lakes, Inc., Arden Hills, MN)].

### Activity Monitoring Tags

Data from the CowManager tags indicated that MIN heifers spent more time (*P* < 0.0001; Table 6) eating compared to CON with the least amount of min/d eating being observed by the NRG heifers. Eating behavior can be defined as when a cow had eating jaw movements and the muzzle was in close contact with the ground (Nielsen, 2013; Pereira et al., 2018). Furthermore, NRG heifers spent more time (*P* < 0.0001) ruminating compared with CON and MIN heifers. Pereira et al. (2018) defined rumination as when a cow was standing, walking, or lying and the cow regurgitated a bolus and chewed the cud while moving her head and jaw in a circular motion and then swallowing the masticate. Heifers consuming NRG supplement spent more time (*P* < 0.0001) not being active compared with MIN (180.8 min/d) being intermediate and CON (175.6 min/d) heifers spending the least amount of time not active. Conversely, CON heifers were most active (234.7 min/d; *P* < 0.0001) compared to MIN being intermediate and NRG heifers being the less active compared to other treatments (200.5 and 187.6 min/d, respectively). Mineral and CON heifers spent a similar amount of time being highly active (141.1 and 141.4 min/d, respectively), with NRG heifers spending 20 more minutes being highly active (165.3 min/d; *P* < 0.0001) compared with other treatments. Heifers reported herein were moving throughout the pasture as a herd of 60 cattle, which may explain the high level of activity reported from the 13 NRG heifers as they competed for supplement from the 2 feeder spaces in the MCCC units. The CowManager system has been validated for rumination and activity measures in dairy cattle kept in freestalls (Bikker et al., 2014), or grazing (Pereira et al., 2018), and with feedlot cattle in confinement (Wolfger et al., 2015). However, there is paucity of data related to beef cattle managed in extensive grazing scenarios.

**Table 6.** Activity of heifers monitored using CowManager ear tags while grazing native range and access to mineral (MIN) or energy with mineral (NRG) supplements

	Treatment^1^				
Parameter^2^, min/d	CON	MIN	NRG	SEM	*P*-value
Eating	535.3^b^	572.6^c^	497.3^a^	5.57	<0.0001
Ruminating	352.2^a^	345.4^a^	393.3^b^	4.13	<0.0001
Not active	175.6^a^	180.8^b^	198.7^c^	1.83	<0.0001
Active	234.7^c^	200.5^b^	187.6^a^	5.04	<0.0001
Highly active	144.4^b^	141.1^a^	165.3^c^	1.17	<0.0001

^a,b^Means within a row with a different superscript differ (*P* ≤ 0.05).

^1^Treatments include: CON (*N*= 29), no access to feed supplements; MIN (*N* = 18), free choice access to mineral supplement [Purina Wind & Rain Storm All-Season 7.5 Complete (Land O’Lakes, Inc., Arden Hills, MN)]; NRG (*N* = 13), free choice access to energy supplement [Purina Accuration Range Supplement 33 with added MIN (Land O’Lakes, Inc., Arden Hills, MN)].

^2^Parameters from the CowManager system (CowManager B.V, the Netherlands) are collected continuously and each minute is classified into behavioral categories (i.e., “eating”, “ruminating”, “not active”, “active”, and “highly active”) using a proprietary model.

The retrospective evaluation of estrus alerts generated via the CowManager system revealed that 16 of 28 heifers (57%) confirmed pregnant via ultrasound were incorrectly identified as displaying some type of estrus behavior (two reported as in heat, 11 reported as potential, and three reported as suspicious). If producers were using this technology for estrus detection in a pasture setting, additional confirmation of estrus behavior would be important to consider for use in AI breeding. Additional resources such as estrus detection patches to determine heat state (Hill et al., 2014) or visual observations may be beneficial to have as additional or alternative means to validate estrus alerts generated by the CowManager system. Multiple estrus detection technologies [Cowmanager SensOor (Agis Automatisering, Harmelen, the Netherlands), HR Tag (SCR Engineers Ltd., Netanya, Israel), Ice-Qube (IceRobotics Ltd., Edinburgh, UK), DVM bolus (DVM Systems, LLC, Greeley, CO) and The Track a Cow (Animart Inc., Beaver Dam, WI)] have been validated on dairy cattle (Dolecheck et al., 2015), in which all activity measures increased during estrus compared with animals not in estrus. Additionally, these validations have analyzed correlations among different technologies on the same animal or comparing human observations of estrus or activity measures. Nevertheless, the current study did not evaluate additional technologies to compare estrus activity, did not have visual observation, and was not evaluated as an indicator of estrus for first-service AI.

The retrospective evaluation of health alerts generated via the CowManager system revealed 34 out of 60 heifers generated 146 health alerts, but only 3 heifers of the heifers initiating an electronic health alert needed clinical treatment (each for symptoms associated with foot rot). Animal care staff identified an additional nine heifers that required treatment for foot rot for which no health alert was generated by the CowManager system. On a beef operation, to minimize loss of health costs, it is essential to observe behavioral and physiological changes as early as possible. Therefore, utilizing automatic monitoring of activity and feeding behavior can provide producers and researchers with an early warning tool (Bikker et al., 2014). However, utilizing sensor monitoring systems requires the producer or researcher to rely on their intuition and experience (herdsmanship) when interpreting the available information to make intervention decisions (Rutten et al., 2013). These technologies provide benefits to producers and researchers when they can easily monitor cattle without disturbing natural behavior; however, technologies must accurately and easily quantify behavioral and physiological parameters (Senger, 1994). Some monitoring systems for rumination and estrus have been validated with high accuracy in dairy cattle (Bikker et al., 2014; Dolecheck et al., 2015); however, validation of rumination collars in beef cattle resulted in underestimated rumination times (Goldhawk et al., 2013). More research is required to refine the algorithms for estimation of activity and behavioral parameters when used in beef cattle grazing extensive pastures. Having a more refined algorithm for grazing beef cattle may enhance monitoring of estrus and health events in beef cattle production systems.

## CONCLUSIONS

Our objectives were to develop a Mobile Cow Command Center (MCCC) for monitoring heifers on native range specifically to 1) examine the relationship between mineral and energy supplementation on intake, liver mineral concentrations, and metabolites and 2) examine activity, reproductive, and health behavior. The MCCC units were deployed successfully and serve as portable units that use solar power to run individual components and upload data to cloud-based data acquisition platforms. Though not all assigned heifers voluntarily consumed feed from electronic feeders, the SmartFeed units were able to control intake of individual animals assigned to different treatments in a group pasture scenario. Our results clearly show the ability of the electronic feeders to control intake for precision feeding of individuals in extensive group managed scenarios. The potential exists to develop targeted management strategies for cattle with distinct nutrient needs (i.e., high and low body condition scores or mixed groups of cows and heifers) while being managed in common pastures. Furthermore, the CowManager system was able to detect divergence in highly active behavior but also reported many false health and estrus-related alerts. Technological solutions must be made that incorporate algorithms developed using training sets originating from pasture-based beef cattle for optimal utility, performance, and adoption in beef cattle production systems.
